# Nocebo effect in multiple system atrophy: systematic review and meta-analysis of placebo-controlled clinical trials

**DOI:** 10.1007/s10072-021-05758-2

**Published:** 2022-01-01

**Authors:** Zi-Xuan Wang, Nan-Nan Zhang, Hai-Xia Zhao, Jie Song

**Affiliations:** 1 Department of Geriatrics, the Affiliated Hospital of Qingdao University, Qingdao, 266071 China; 2 Institute of Neuroregeneration and Neurorehabilition, Qingdao University, Qingdao, 266071 China; 3 Department of Neurology, the Third People’s Hospital of Qingdao, Qingdao University, Qingdao, 266000 China

**Keywords:** Nocebo effect, Placebo, Multiple system atrophy, Randomized controlled trials, Meta-analysis

## Abstract

**Background:**

Nocebo effect is prevalent among neurological diseases, resulting in low adherence and treatment outcome. We sought to examine the nocebo effect in randomized controlled trials (RCTs) in multiple system atrophy (MSA).

**Methods:**

We searched RCTs in MSA from Medline since September, 2021. RCTs for drug treatment conducted in adult MSA patients with more than 5 cases in each treatment arm were included. We assessed the number of dropout due to placebo intolerance. We also did a symptomatic/disease-modifying subgroup analysis based on two different treatment purposes. The STATA software was used for statistical analysis. Overall heterogeneity was assessed using the Cochran *Q* and *I*^2^.

**Results:**

Data were extracted from 11 RCTs fulfilling our search criteria. Of 540 placebo-treated patients, 64.2% reported at least one adverse event (AE) and 7.5% reported dropout because of AEs. The chance of dropping out because of an AE and experiencing at least one AE did not differ between placebo and active drug treatment arms. Besides, the pooled nocebo dropout rate in the symptomatic subgroup was similar to that of the disease-modifying subgroup.

**Conclusion:**

In MSA RCTs, nocebo dropout rate was not at a low level among neurological disorders. Nocebo effect was an important reason of dropout because of AE in placebo and active drug treatment arms. Different treatment purposes may not influence nocebo effect.

## Introduction

The psychosocial context can play an essential role in most medical treatments. Positive physiological or psychological effects induced by the treatment context are referred to as well- known placebo effects. Placebo effect has impact on therapeutic response, adherence, and quality of life [1, 2]. On the other hand, the negative effects the treatment context induces are defined as nocebo effects. Nocebo effect refers to the experience of AEs from administration of an inert substance such as placebo. This phenomenon is influenced by patients’ anticipations, previous experiences to medication, awareness of drug side-effect profiles, psychological factors including stress and anxiety, and the interactions between clinicians and patients [3]. Nocebo effects interfere negatively with medical and treatment outcomes and physicians should be able to propose strategies for preventing or even overcoming their influence so as to optimize treatment outcomes [4].

Nocebo effect is prevalent among neurological diseases and a variability in the magnitude of nocebo effect has been demonstrated in various neurological disorders [5]. To date, there is sparse reporting of nocebo in MSA drug treatment, with only a RCT of MSA [6] having been investigated. The aim of our study is to estimate the frequency and strength of nocebo effects in RCTs of MSA. We also sought to examine whether different treatment purposes influenced nocebo effect. In consistent with the former studies, the dropout rate due to placebo-related AE was also used as measure of nocebo effect in our study.

## Methods

This systematic review and meta-analysis was conducted according to the Preferred Reporting Items for Systematic Reviews and Meta-Analyses (PRISMA) guidelines [7].

### Data source search strategy

A computer-based PubMed literature search was conducted on 28th Sep, 2021. We used two Medical Subject Headings (MeSH) terms. Term A was “placebo” and Term B was “multiple system atrophy” or “MSA.” Limitations were set to specify language to be English.

### Study selection

We included the studies if they met the following criteria: (1) referred specifically to MSA; (2) referred specifically to humans; (3) RCTs; (4) pharmaceutical studies; (5) there was a placebo arm; (6) each treatment arm had at least 5 patients; (7) adult participants; and (8) detailed data of drop-outs in each treatment arm were available in the report; (9) excluding reviews, letters without original data, editorials; (10) when there was more than one publication from the same studied population, only data from the most comprehensive report was included in our analysis and the remaining were excluded.

### Data extraction and quality assessment

Two reviewers independently performed the search, reviewing all articles and collecting data. Items were extracted from each study and the extracted information included: information on the article identification; year of publication; evaluation period; total number of subjects; number of placebo-treated subjects; total number of placebo deaths; number of placebo-treated subjects who dropped out because of AEs; number of placebo-treated subjects who dropped out for other reasons; number of male subjects treated with placebo; mean age of placebo-treated subjects; number of active drug-treated subjects; total number of active drug deaths; number of active drug-treated subjects who dropped out because of AEs; number of active drug-treated subjects who dropped out for other reasons; number of male subjects treated with active drug; mean age of active drug-treated subjects; active drug; disease-modifying drug or symptomatic drug; route of drug administration and geographic location. The authors independently evaluated each of the included studies according to modified JADAD scale [8]. RCT with a JADAD score ≥ 4 was considered a high-quality study.

### Statistical analysis

The meta-analysis was conducted using STATA 15.0 (Copyright 1985–2017 Stata Corp LP). We calculated the pooled dropout rates because of any AEs for each group and its 95% confidence intervals, as well as the pooled AE rates for each group and its 95% confidence intervals. We choose odds ratios (ORs) and 95% confidence intervals (CIs) as the appropriate parameters to evaluate the dichotomous outcomes, such as the dropout rate. Overall heterogeneity was assessed using the Cochran *Q* test (*p* value > 0.05 indicates lack of heterogeneity among studies) and *I*^2^ value (values < 50% as low heterogeneity). The fixed-effect model was applied if there was no or low heterogeneity. Data were analyzed using a random effects model if there was high heterogeneity. Pooled ORs for comparison between the two arms were estimated as illustrated in the forest plots. The Egger’s test was used to assess the presence of asymmetry in the funnel plots (*p* value > 0.05 indicates no publication bias).

## Results

**Fig. 1:**
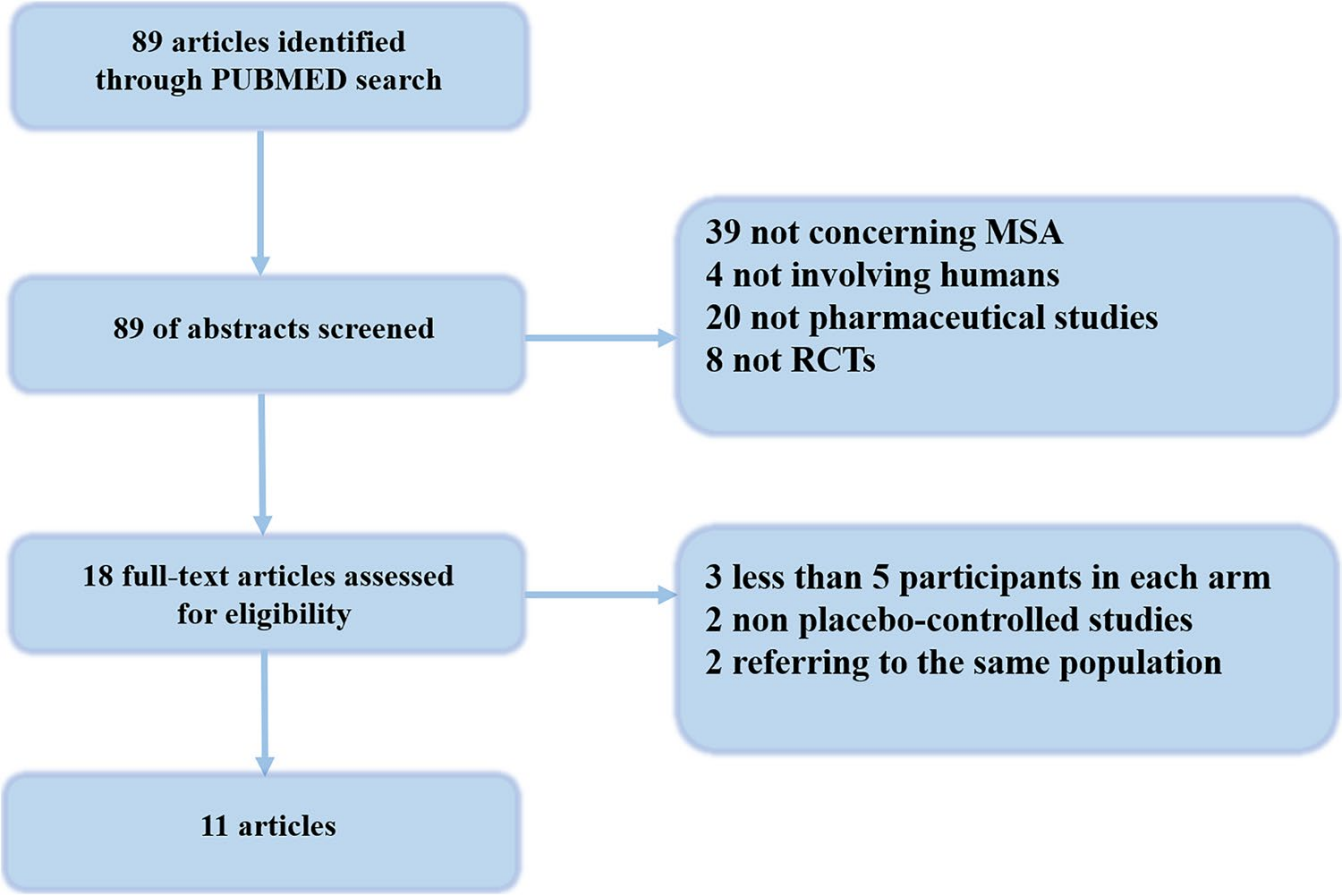
The PRISMA chart showing study screening process

The process of the article selection is presented in Fig. 1. After the application of our search strategy, we found that eleven RCTs [9–19] published between 1996 and 2021, that met our selection criteria. The included RCTs have a JADAD score of 4 or greater. Five hundred forty placebo-treated MSA patients and 558 active drug-treated MSA patients were included. Data details of the included studies are presented in the Table 1.

**Table 1:** The characteristics of the included studies

Study	Active drug	Route of drug administration	Purpose of treatment	Placebo-treated subjects							Active drug-treated subjects						
				Country	Number	Subjects of AE	Deaths	Dropouts due to AE	Males (%)	Mean age (years)	Number	Subjects of AE	Deaths	Dropouts due to AE	**Males** (%)	Mean age (years)	JADAD
Rascol et al., 2021 [19]	Fluoxetine	Oral	Symptomatic	France	41	38	2	5	58.5	63.5 ± 8.1	40	39	3	8	65.0	63.1 ± 7.8	7
Jung Lee et al., 2020 [18]	IMP	Oral	Disease-modifying	South Korea	25	15	2	3	60.0	59.8 ± 6.8	30	20	1	5	50.0	59.9 ± 6.8	7
Meissner et al., 2020 [17]	Vaccine	Subcutaneous	Disease-modifying	France	6	6	0	0	83.3	66.0	24	23	3	2	58.3	61.0	7
Levin et al., 2019 [16]	EG	Oral	Disease-modifying	Germany	45	14	2	5	58.0	64.0	47	18	4	3	62.0	60.0	7
Low et al., 2017 [14]	Rifampicin	Oral	Disease-modifying	USA	50	31	5	3	68.0	61.1 ± 9.2	50	31	2	3	44.0	60.9 ± 7.8	7
Poewe et al., 2015 [15]	Rasagiline	Oral	Disease-modifying	12 countries	90	67	2	7	57.0	65.1 ± 8.6	84	68	3	14	58.0	64.9 ± 8.5	7
Dodel et al., 2010 [13]	Minocycline	Oral	Disease-modifying	Germany	31	NA	2	6	39.0	61.0 ± 8.0	32	NA	3	8	53.0	63.0 ± 7.0	6
Bensimon et al., 2009 [12]	Riluzole	Oral	Disease-modifying	Europe	201	NA	0	0	55.8	62.6 ± 8.1	203	NA	0	0	53.8	61.9 ± 8.5	7
Holmberg et al., 2007 [11]	hGH	Injected	Disease-modifying	Europe	21	19	2	1	71.4	62.0 ± 7.4	22	21	3	3	50.0	58.4 ± 9.0	4
Friess et al., 2006 [10]	Paroxetine	Oral	Symptomatic	Germany	11	0	0	0	36.4	67.9 ± 5.0	9	1	0	1	66.7	62.7 ± 10.4	5
Botez et al., 1996 [9]	AH	Oral	Symptomatic	Canada	19	NA	0	1	40.0	40.0 ± 2.0	17	NA	0	2	33.3	40.0 ± 2.0	5

*IMP*, inosine 5′-monophosphate; *vaccine*, α-synuclein vaccines PD01A and PD03A; *EG*, epigallocatechin gallate; *hGH*, human growth hormone; *AH*, amantadine hydrochloride; *NA*, not applicable

## Dropouts in placebo and active drug arm

**Fig. 2:**
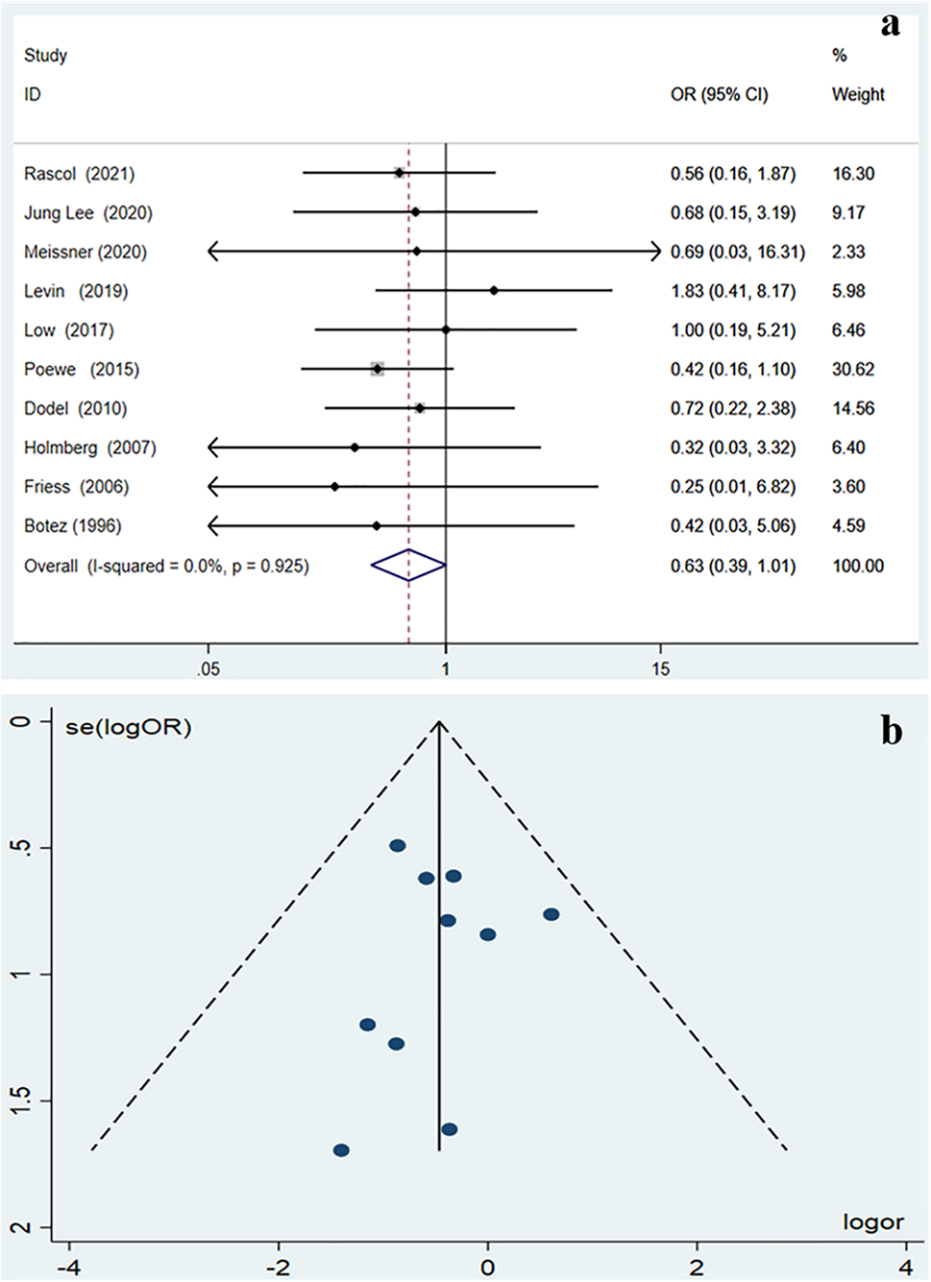
**a** Meta-analysis results as illustrated in the forest plot regarding the percentage of patients who dropped out because of an adverse event. **b** Meta-analysis results as illustrated in the funnel plot regarding the percentage of patients who dropped out because of an adverse event

The pooled estimate of dropout rate because of AEs other than death in placebo-treated patients and active drug-treated patients was 7.5% (95% *CI* = 3.0–13.7%) and 11.8% (95% *CI* = 5.1–20.8%) respectively. One study [12] reported no dropouts because of AEs in both study arms and other ten studies were included in the final meta-analysis. As illustrated in the forest plot (Fig. 2a), the chance of dropping out because of AEs other than death did not differ between placebo and active-drug treatment arms (*OR* = 0.626, 95% *CI* = 0.389–1.007, *p* = 0.053). There was no heterogeneity among the included studies (*p* = 0.925, *I*^2^ = 0%). As shown in the funnel plots (Fig. 2b), there was no publication bias among the included studies (Egger test: *z* =  − 0.45, *p* = 0.655). Besides, 17 deaths occurred in placebo-treated patients of 7 studies and 22 deaths occurred in active drug-treated patients of 8 studies (Table 1). All of the deaths were considered unrelated to active treatment, except for those not specified in the articles.

## Adverse events in placebo and active drug arm

**Fig. 3:**
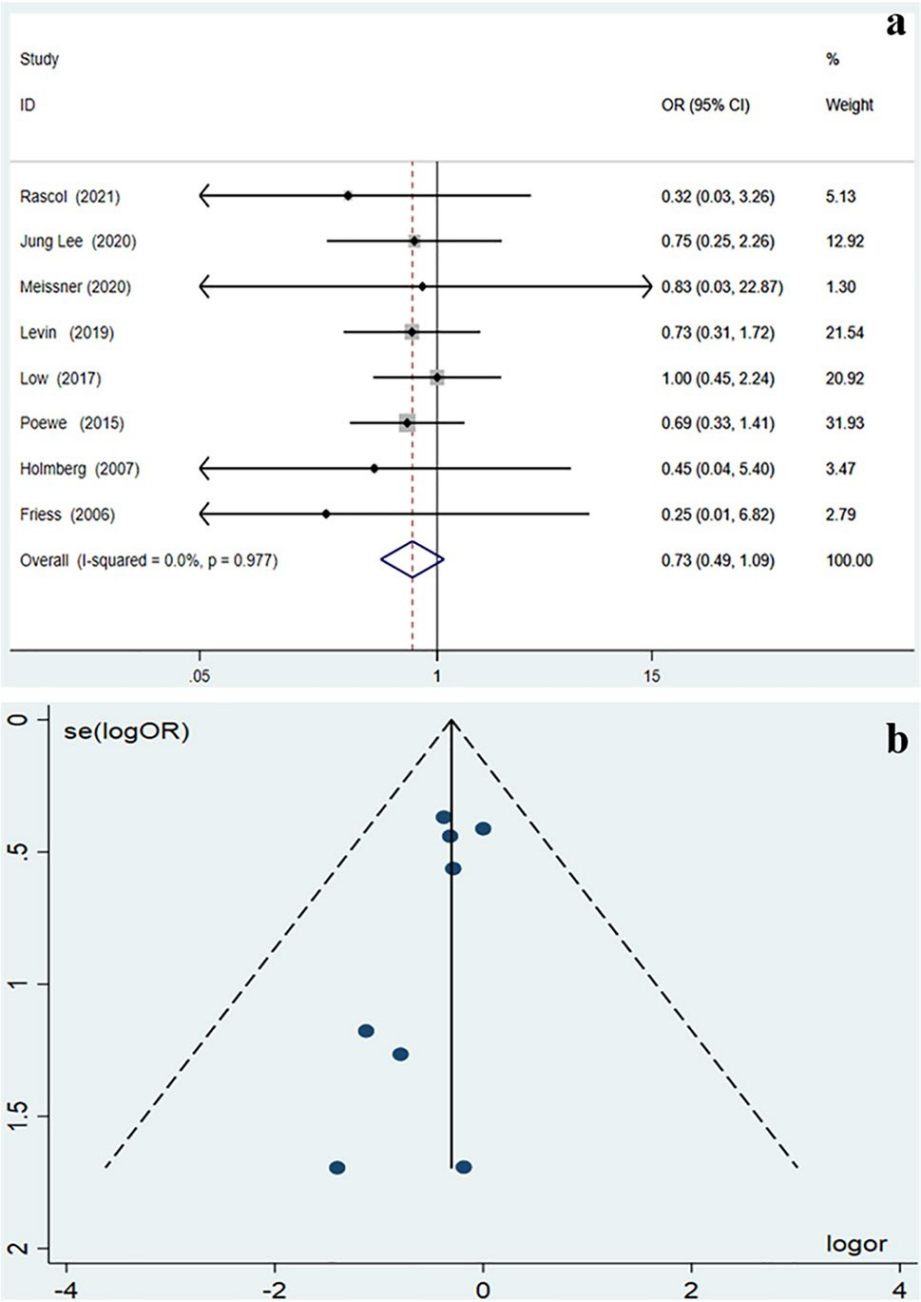
**a** Meta-analysis results as illustrated in the forest plot regarding the percentage of patients who experienced at least one adverse event. **b** Meta-analysis results as illustrated in the funnel plot regarding the percentage of patients who experienced at least one adverse event

Only eight of the studies [10, 11, 14–19] reported in detail the exact number of patients experiencing at least one AE in the study groups. The pooled estimate of the percentage of placebo treated patients with at least one AE was 64.2% (95% *CI* = 43.2–82.6%), compared to 72.4% (95% *CI* = 53.4–88.0%) for active drug treated patients. As illustrated in the forest plot (Fig. 3a), the chance of experiencing at least one AE did not differ between placebo and active drug treatment arms (*OR* = 0.732, 95% *CI* = 0.490–1.092, *p* = 0.126). There was no heterogeneity among the studies included in the meta-analysis (*p* = 0.977, *I*^2^ = 0.0%). As shown in the funnel plots (Fig. 3b), there was no publication bias among the included studies (Egger test: *z* =  − 0.74, *p* = 0.458).

### Dropouts for MSA patients with two different purposes of experimental treatment

The MSA patients included in 11 studies were classified into two subgroups according to different purposes of experimental treatment. Three of the studies were symptomatic and eight of the studies were disease-modifying (Table 1). In the two subgroups, the chance of dropping out because of AEs other than death did not differ between placebo and active-drug treatment arms (symptomatic subgroup: *OR* = 0.484, 95% *CI* = 0.173–1.358, *p* = 0.168; disease-modifying subgroup: *OR* = 0.672, 95% *CI* = 0.392–1.149, *p* = 0.146). There was also no heterogeneity among the studies included in two subgroups (symptomatic subgroup: *p* = 0.895, *I*^2^ = 0.0%; disease-modifying subgroup: *p* = 0.775, *I*^2^ = 0.0%). Moreover, the pooled nocebo dropout rate in the symptomatic subgroup (9.2%, 95% *CI* = 3.7–16.7%) was similar to that of the disease-modifying subgroup (7.5%, 95% *CI* = 2.3–15.4%).

## Discussion

This meta-analysis of 11 RCTs showed the chance of dropping out because of AE other than death and experiencing at least one AE did not differ between placebo and active drug treatment arms. The dropout rates were similar across the study arms and independent of the study arm to which they belonged. This indicates that nocebo effect is an important reason of dropout because of AEs other than death in placebo and active drug treatment arms in MSA RCTs.

Nocebo varies significantly among neurological diseases. Weighed against other neurological disorders investigated with identical methodology, comparable to those found in fibromyalgia (nocebo dropout rate 9.5%, 95% *CI* = 8.3–10.9%; nocebo AE rate 67.2%, 95% *CI* = 51.0–81.5%) and chronic inflammatory demyelinating polyneuropathy (CIDP) (nocebo dropout rate 2.1%, 95% *CI* = 0.3–7.3%; nocebo AE rate 42%) [20], the nocebo dropout rate and nocebo AE rate were not at a low level in MSA among neurological disorders and close to those found in Alzheimer’s disease (AD) (nocebo dropout rate 6.6%, 95% *CI* = 5.3–8.4%, nocebo AE rate 57.8%, 95% *CI* = 50.1–66.7%) [21] and Parkinson’s disease (PD) (nocebo dropout rate 8.8%, 95% *CI* = 6.8–11.5%, nocebo AE rate 64.7%, 95% *CI* = 53.6–74.4%) [22]. In terms of neurobiological mechanisms, high nocebo responses were associated with activation of cholecystokinin (CCK) system, hyperactivity of the hypothalamic–pituitary–adrenal (HPA) axis, deactivation of dopamine (DA), and opioid release. Nocebo system involves different brain regions and can modulate the outcome of a given therapy in a negative way [23]. MSA is a neurodegenerative disorder characterized by prominent autonomic failure associated with parkinsonism, cerebellar ataxia, or both [24]. Patients with MSA showed defective DA and opioids systems [25, 26]. MSA patients are affected in some regions of the brain where nocebo effects come into play [27, 28]. Thus, as we found, powerful nocebo should be expected in MSA. Furthermore, similarities in nocebo rates among the neurodegenerative diseases enhance the theory that nocebo rates may vary depending on the pathophysiology and the pathology of the disease [23].

A treatment may be investigated with a potential disease-modifying purpose, others as a symptomatic drug. From the conceptual perspective of the nocebo effect, different treatment purposes may influence patients’ expectation towards the experimental drug and the study participation. We did a further subgroup analysis. Our results showed that in the symptomatic subgroup/disease-modifying subgroup, the chance of dropping out because of AEs other than death did not differ between placebo and active-drug treatment arms. Moreover, the pooled nocebo dropout rate in the symptomatic subgroup was similar to that of the disease-modifying subgroup. Our results indicated that nocebo effect did not appear to be affected by different purposes of experimental treatment in MSA RCTs.

## Study limitations

Our results should be interpreted with caution due to the limitations of the design. Firstly, the trail dropouts did not accurately reflect the nocebo effects and its severity. Because it is difficult to accurately attribute AEs in the placebo group to drug administration or a consequence of disease worsening or non-specific symptoms, dropouts used as measure of nocebo effect exist deficiency. Secondly, this meta-analysis was performed using only eleven RCTs, which involved pharmacotherapy in MSA and met our stringent inclusion criteria. Some MSA-related RCTs, which involved other diseases such as neurogenic orthostatic hypotension and pure autonomic failure, were not included in our analysis. Therefore, our estimates may not fully reflect the extent of nocebo effect in MSA patients.

## Implications for clinical trials and clinical practice

Clinicians should be aware of the nocebo phenomenon and attempt to reduce nocebo effect in clinical trials and clinical practice [4]. A number of accepted strategies minimizing nocebo effect, including creating a good physician–patient relationship, increasing empathic attitudes, exposing information suitably, decreasing expectations of AEs, still applicable to MSA clinical trials and clinical practice. It is also useful for clinicians to acknowledge that reported nocebo AE rates and nocebo dropout rate are not low in RCTs for MSA. A similar dropout rate in both placebo and active-drug arms implicates that most of the dropout in RCTs of MSA are due to nocebo effect. The causal relationship between AEs and drugs should be analyzed prudently and be achievable with a combination of detailed safety reports and structured AE assessments. Clinicians should rely more on objective investigations than on subjective symptoms reported by patients. Furthermore, because different purposes of treatment did not appear to be relevant to nocebo effect, clinicians did not worry about the impact of treatment purpose on nocebo effect in MSA RCTs.

## Conclusion

In RCTs for MSA, 64.2% of the participants reported AEs related to nocebo and 7.5% of the participants reported dropout related to nocebo. With dropout rate as measure of nocebo effect, the total patients participating in those RCTs reported similar dropout independently of the study arm they belonged, which suggests some AEs arise from the anticipated pharmacological effects of the drug other than active drug itself. Nocebo effect was the important reason of dropout because of AE in the placebo and the active drug groups and is an important factor for treatment adherence in MSA patients during clinical trials and in clinical practice. Furthermore, the nocebo effect does not appear to be affected by different purposes of experimental treatment in MSA RCTs.
